# DEDB: a database of *Drosophila melanogaster *exons in splicing graph form

**DOI:** 10.1186/1471-2105-5-189

**Published:** 2004-12-07

**Authors:** Bernett TK Lee, Tin Wee Tan, Shoba Ranganathan

**Affiliations:** 1 Department of Biochemistry, National University of Singapore, Singapore; 2 Biotechnology Research Institute, Macquarie University, Sydney, Australia

## Abstract

**Background:**

A wealth of quality genomic and mRNA/EST sequences in recent years has provided the data required for large-scale genome-wide analysis of alternative splicing. We have capitalized on this by constructing a database that contains alternative splicing information organized as splicing graphs, where all transcripts arising from a single gene are collected, organized and classified. The splicing graph then serves as the basis for the classification of the various types of alternative splicing events.

**Description:**

DEDB  is a database of *Drosophila melanogaster *exons obtained from FlyBase arranged in a splicing graph form that permits the creation of simple rules allowing for the classification of alternative splicing events. Pfam domains were also mapped onto the protein sequences allowing users to access the impact of alternative splicing events on domain organization.

**Conclusions:**

DEDB's catalogue of splicing graphs facilitates genome-wide classification of alternative splicing events for genome analysis. The splicing graph viewer brings together genome, transcript, protein and domain information to facilitate biologists in understanding the implications of alternative splicing.

## Background

The completion of the draft sequence of the *Drosophila melanogaster *genome in March 2000 [1,2] and the availability of quality annotations by FlyBase in 2002 [3] presents an excellent opportunity for the study of alternative splicing. Although the annotations themselves provide an insight to the amount of alternative splicing, they do not provide any classification of the types of alternative splicing events present. Different forms of alternative splicing have different biological bases and the classification of alternative splicing events is critical for further work in deciphering the regulatory controls that govern these processes. To this end, we transformed all known gene structure information obtained from the genome annotations into splicing graphs based on the approach first proposed by Heber et al. in 2002 [4]. We then created simple but robust rules for classifying the splicing graphs into various alternative splicing events. The rules created allows for the detection of multiple forms of alternative splicing within the same gene. To facilitate the assessment of the impact of alternative splicing on the protein product in particular with respect to the domain organization of the protein, Pfam [5] domains were mapped onto the transcripts using HMMER [6]. All these data were then loaded into DEDB (*Drosophila melanogaster *Exon Database) [7]. To aid in visualizing these splicing graphs, a web-based splicing graph viewer was also developed. The splicing graph viewer integrates gene structure, transcript, protein and domain information into an easily understandable interface that is viewable with any current web browser. The splicing graphs as well as the alternative splicing event classifications are available for download as XML files. A XML schema is available for parsing and validation of the XML files.

## Construction and content

### Data source

*Drosophila melanogaster *genome annotations (release 3.2) were obtained from FlyBase [8] as Game XML files. Gene structure information including the location of the transcript, the start and end positions of each exon that make up the transcript and the protein coding region were parsed out, checked for consistency and then loaded into a relational database (MySQL). Pfam HMM models were retrieved from Pfam release 12 and used as the database for the hmmpfam program (part of HMMER) to search the transcript protein sequences for structural domains, with an expectancy values of less than 0.001. The results of the search were parsed, mapped onto the protein sequence and imported into the database.

### Construction of the splicing graphs

**Figure 1:**
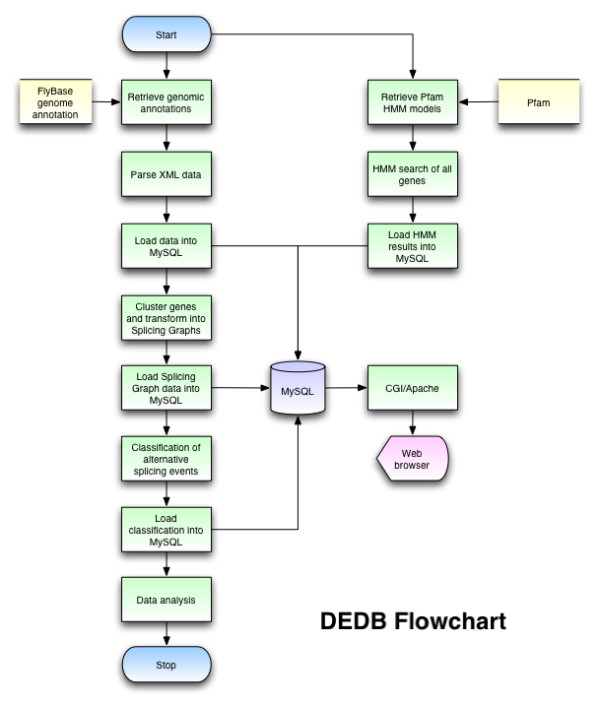
**Flowchart depicting the process used to generate DEDB. **Processes and data sources are coloured green and yellow respectively. The main data store is a MySQL server and the data housed is exposed to users using HTML pages and CGI (Common Gateway Interface) served by an Apache web server.

The transcripts contained in the database were retrieved and clustered on the basis that they occupy overlapping genomic positions. Splicing graphs are then constructed using these clusters of transcripts. In each cluster, exons and introns having identical start and end positions are merged into nodes and connections respectively. The nodes are then linked via connections to form the complete splicing graph that is loaded into the database. In cases where the transcripts are located on the negative strand, the entire splicing graph is reverse complemented so that all the splicing graphs contained in the database have sense strand nucleotide sequences. These steps are graphically depicted in Figure 1. The result of this process generated 13,222 splicing graphs of which 2,646 are alternatively spliced. Details of the contents of the database are found in Table 1 and on the website via the "Stats" link.

### Classification of alternative splicing

**Figure 2:**
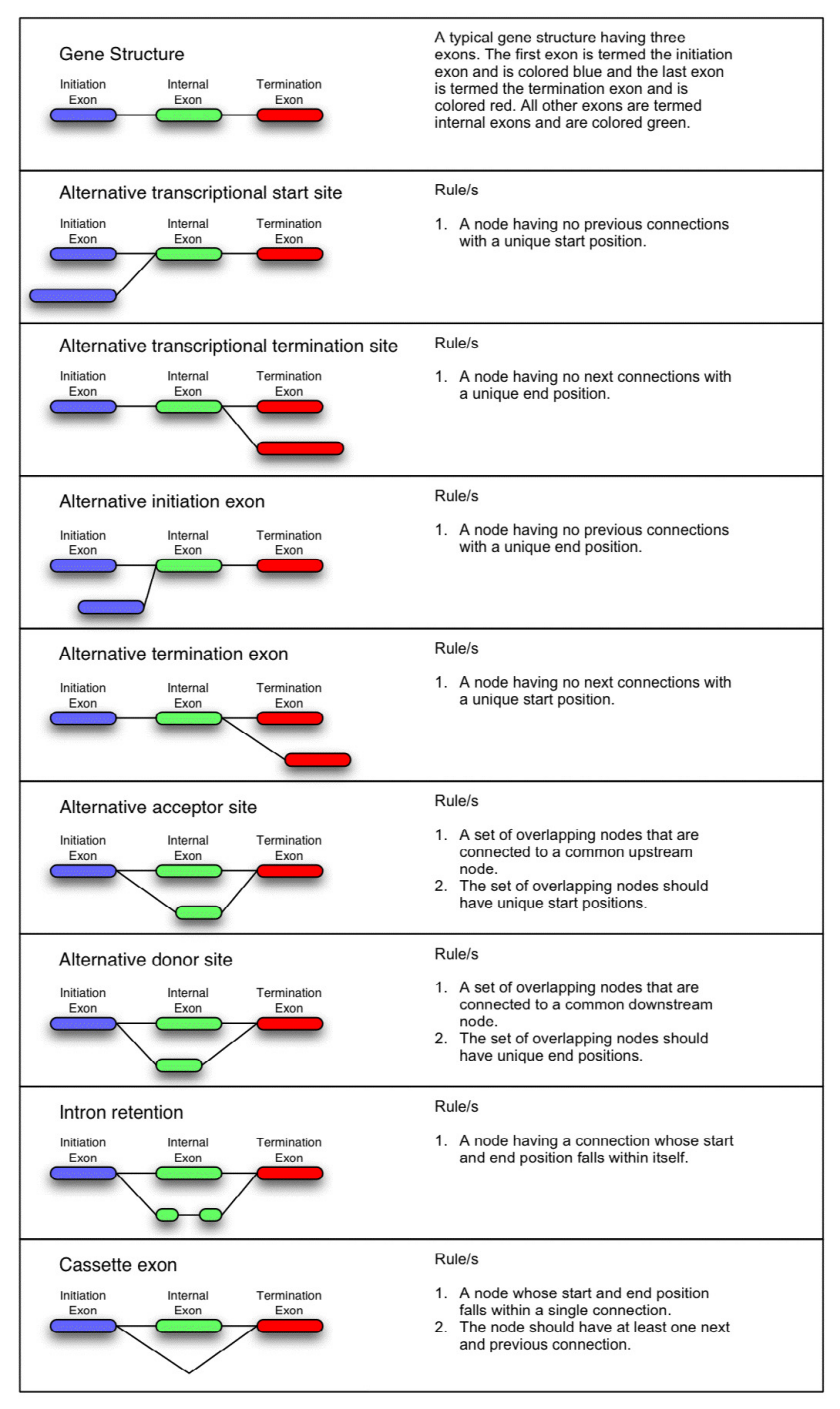
**Rules used to classify splicing graphs in DEDB. **Initiation, internal and termination exons are colored blue, green and red respectively. Each of the type of alternative splicing event and gene structure event is depicted as a single boxed row. Within each boxed row, a graphical illustration of the gene structure is shown on the left together with the textual rules on the right.

Rules are then derived to detect specific alternative splicing events as shown in Figure 2 (details and examples of the rules are available on the website). Apart from the classical alternative splicing events like cassette exons, intron retention, alternative donor sites and alternative acceptor sites, we have also elected to classify other gene structure events like alternative transcriptional start/termination sites as well as alternative initiation/termination exons. The reason for the existence of the alternative initiation/termination exon categories is due to the fact that the 5' and 3' ends of the transcripts are usually difficult to determine experimentally and are thus less accurate. Therefore, any differences in the start and end of the transcripts could be simply due to the sequencing difficulties. The inclusion of the alternative initiation/termination exons category is an attempt to circumvent this problem as alternative initiation/termination exons (which are classified based on the end position of initiation exons and the start position of termination exons) are unaffected by the sequencing difficulties and thus represent true alternative exons. Alternative transcriptional start/termination sites, however, are dependent on sequencing results and provide a means of classifying gene segments with differences in the start positions of initiation exons and the end positions of termination exons, with a view to updating entries in this category, when the 5' and 3' ends of these transcripts are determined accurately. These rules were then used on all the splicing graphs and the detected alternative splicing events loaded into the database.

### Access

The database together with the splicing graph viewer is freely available at . Users can query the database using FlyBase gene names, FlyBase Gene Symbols, Pfam Accession Numbers or Pfam Identifiers via the query page. Users can also query the database using BLAST [9] searches. This is particularly useful if one wishes to know the *Drosophila melanogaster *homology together with alternative splicing information of a particular gene. Lists of splicing graphs for the various types of alternative splicing events are also provided on the website for users who are interested in a certain type of alternative splicing. For users who wish to use large subsets of the data, they can download the XML files available from the same site. To aid parsing and validation of the XML file, a XML schema is available. DEDB can also be accessed via links on Flybase gene records, under the external database links section. Correspondingly, the DEDB Splicing Graph Viewer provides links back to FlyBase Gene and Annotation records, where experimental evidence for the gene structure has also been provided. Basic statistical analysis of the database can be found at the DEDB website .

### Splicing graph viewer

**Figure 3:**
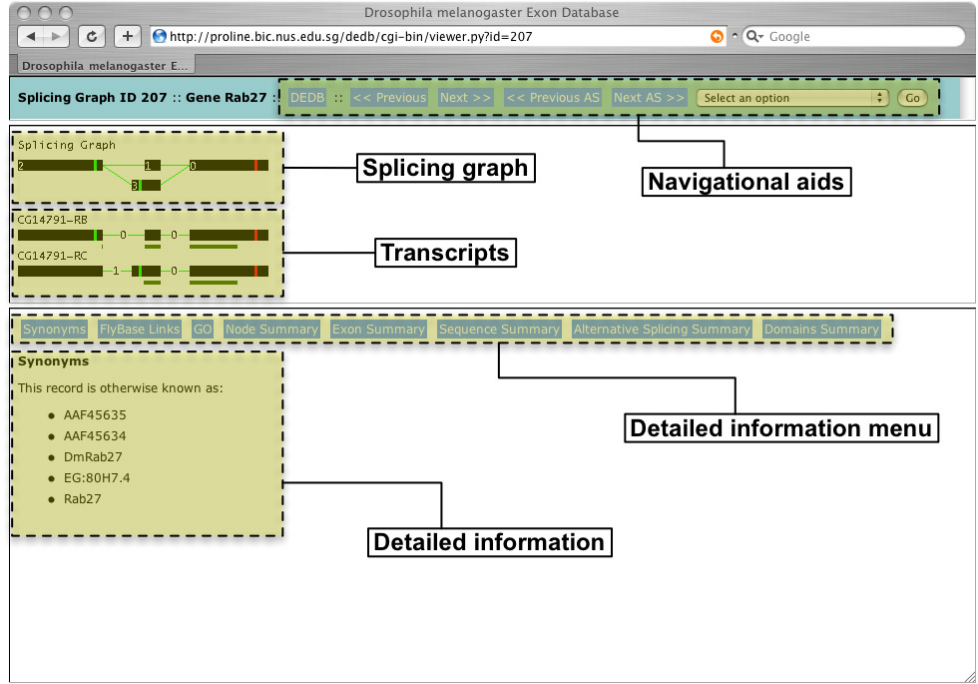
**Screenshot of DEDB Splicing Graph Viewer. **The viewer is divided into three frames. The top frame contains the navigational aids to allow users to quickly locate the splicing graph of interest. The middle frame shows the splicing graph together with all the transcripts used to generate the splicing graph. The splicing graph and the transcripts are interactive allowing users to click on them for more information in the bottom frame. The bottom frame shows textual information about the splicing graph, which are selectively displayed by either clicking on interactive elements in the middle frame or by clicking on the row of buttons in the bottom frame.

The splicing graph viewer consists of HTML pages created using a series of Python CGI (common gateway interface) scripts served by the Apache web server. The splicing graph viewer (Figure 3) is a three frame HTML page that shows the splicing graph in the center frame with detailed textual information in the bottom frame and navigation aids in the top frame (details elaborated on the website). The content is organized such that generalized information is provided to the users initially, permitting users to quickly zoom in on the information they need by clicking on an item of interest. The splicing graph shown in Figure 3 has alternative acceptor sites, where nodes 1 and 3 are alternatively used. Each node here can be selected and the corresponding node information, with sequence details is then dynamically displayed in the bottom frame. The transcripts leading to this splicing graph are depicted below the splicing graph. The connections in each transcript represent introns, which can also be selected to obtain intron-specific information. The rationale for providing the transcripts is because not all the paths in the splicing graphs are expressed transcripts, so the connections depicted in the splicing graph view are transcript-specific and thus not selectable by DEDB users. The provision of schematic diagrams of the transcripts along with the splicing graph provides the user with knowledge of which transcripts are expressed/detected. Below each transcript, links to structural domains, are shown as thin lines wherever available, linked to detailed information derived from Pfam and viewable on the bottom frame. The sequences displayed by clicking on the nodes in the splicing graph are always shown in the sense orientation to facilitate translation of coding sequences and Pfam mapping, while the exon and intron sequences shown by selecting genomic segments on the transcripts will retain their original orientation identified by chromosomal mapping.

## Utility and Discussion

### Visualization of alternative splicing

By condensing all the various splicing variants into a single graph, where each splicing variant is a path through the graph, users can quickly establish the types and effects of various alternative splicing events present in the gene. Users can quickly pick up bifurcations which denote alternative splicing events far quicker than in the case of the traditional approach of presenting separate schematic representations of each splice variant, where the user has to correlate the splicing patterns from the transcript diagrams, to determine the impact and type of alternative splicing. The classical approach is particularly tedious in cases where the number of splice variants are numerous (for example the *Drosophila moe *gene, splicing graph 916, on the DEDB methodology link) resulting in the user having to correlate large amounts of data to comprehend all the alternative splicing events taking place. The DEDB schematic representation of the splicing graph is different from the one proposed by Heber et al. [4] and implemented in Alternative Splicing Gallery (ASG) [10]. The original representation used single linear block representation of exons connected by lines representing introns and alternative donor and acceptor sites as well as intron retention represented by single blocks. Instead, we have chosen to depict all the exons individually as we felt that this is more intuitive for biologists by making the impact of the alternative splicing more pronounced. Protein sequence details like the start and end of translation as well as detected Pfam domains are presented by the splicing graph viewer. This allows biologists to infer the impact of alternative splicing on the corresponding protein sequences as well as the domain organization. Users can also download FASTA sequences of specific entities like introns and exons for other analysis. Biologists may also be interested in the *Drosophila melanogaster *homology of their gene of interest, which is made possible through a BLAST search on the DEDB query page. The splicing graph of the *Drosophila melanogaster *homology may provide insights into the possible splice variants in the gene of interest. It could also provide information on the level of conservation of alternative splicing between orthologs.

### Classified splicing dataset

The use of splicing graphs allows the creation of simple but robust rules that can detect multiple distinct alternative splicing events within the same gene. Traditional approaches usually require the construction of more complex rules. For example, the detection of a cassette exon in the tradition approach requires that an internal exon be checked against all the introns in all the splicing variants to detect instances where the exon falls within an intron. This process has to be repeated for each exon against all the introns resulting in a long and complex computation. Furthermore as the exon could be found in several splicing variants, the detected cassette exon could be redundant and additional steps have to be taken to remove this redundancy. All of this is avoided by the splicing graph representation, as it is a condensed view of all the various splicing variants arising from a single gene. Classification of the alternative splicing types in *Drosophila melanogaster *would allow users to target specific types of alternative splicing events for analysis. This is useful as the various types of alternative splicing have different biological bases and therefore exhibit different phenotypes. The analysis of these phenotypes will be greatly aided by a set of data that is specific to one form of alternative splicing as provided by DEDB. The availability of a clean datasets of alternative splicing events [11,12] has proved to be useful in providing insights into the phenomenon of alternative splicing [13]. The data available from DEDB would no doubt be useful to many users studying alternative splicing as a major factor leading to complexity in higher eukaryotes.

### Initial analysis

A summary of the alternative splicing events in DEDB is presented in Table 1. Detailed statistical information (general statistics, exon and intron length statistics and motif analysis) are available from the "Stats" page of the website (Lee, Tan and Ranganathan, unpublished results). Note however that the genes models are constructed with far more 5' ESTs than 3' ESTs [3] and the results must be viewed in the light of available experimental EST data.

Of the total of 13,222 genes in DEDB, 2,646 (20%) are alternatively splicing. This is significantly less than the amount of alternative splicing found in higher eukaryotes like humans [14], but sufficient to indicate that alternative splicing is a common phenomenon in *Drosophila melanogaster*. The amount of alternative splicing increases to 24.4% if we consider transcript diversity in the 10,848 multi-exonic genes alone.

Failure of intron definition is more likely to result in intron retention as opposed to exon definition in which case, failure leads to cassette exons. Initial analysis of the DEDB data indicates a bias towards cassette exons (1,228) over intron retention (983) events, so that exon definition is less stringent than intron definition. The short introns in *Drosophila melanogaster *are also thought to result in greater intron definition. The data observed could be due to the splicing machinery adopting a definition model dependent on the length of the intron or exon in question [15]. This is supported by the fact that cassette exons tend to be flanked by introns far longer than the mean value (exon and intron length statistics available via the "Stats" link). The median for the cassette exon length is 150 bp in contrast with the flanking 5' and 3' introns, which are 653 bp and 639 bp respectively. The reverse is also true for intron retention where the median for the intron being retained is 101 bp while the flanking 5' and 3' exons are 163 bp and 261 bp respectively.

Information content analysis indicates that alternative donor and acceptor sites (with mean values of 5.95 and 5.61 bits) possess less information than constitutive sites (9.74 and 8.52 bits respectively; additional data available on website). This observation is consistent with the general notion that alternatively spliced exons exhibit splicing motifs deviating more from the consensus motifs [16]. Cassette exons (CE) and intron retentions (IR) also show lower mean individual information content on both donor (CE: 6.76 and IR: 5.39 bits) and acceptor sites (CE:7.08 and IR: 6.19 bits) as compared to constitutive exons.

The addition of Pfam domain information allows users to assess the impact of alternative splicing events on the proteins generated, enabling correlations not possible with the genome annotations alone.

### Future work

Future work would focus on integrating other relevant information onto the splicing graphs, such as three-dimensional structural information as well as DEDB analysis results. Expansion of the splicing graph representation available in DEDB to other organisms is also underway.

## Conclusions

The data housed in DEDB is organized as splicing graphs, which allows for ease of alternative splicing classifications. This has allowed DEDB to provide clean sets of data containing specific types of alternative splicing events. These specific sets of data could prove useful in understanding the biological basis of alternative splicing because different forms of alternative splicing have different biological basis. The splicing graph viewer provided allows biologists to quickly and intuitively understand the effects of alternative splicing on a gene of interest, thus aiding their research.

## Availability and requirements

The database is available at  suitable for most graphical web browser. XML files of the data contained in the database are also available together with an XML schema to aid parsing.

## Authors' contributions

BTKL carried out the construction of the database as well as the splicing graph viewer. TTW and SR are responsible for the database concept and participated in its design and construction. All authors have read and approved the final manuscript.

## Figures and Tables

**Table 1:** Contents of DEDB. Table showing a breakdown of the contents in the database.

**Item**	**Number**
Total number of transcripts	18,156
Total number of single exonic genes	2,374
Total number of multi exonic genes	10,848
Total number of splicing graphs	13,222
Total number of exons	88,403
Total number of introns	70,247
Total number of nodes	60,744
Total number of connections	46,090
Total number of splicing graphs having alternative splicing events	2,646
Total number of splicing graphs having alternative TSS events	1,696
Total number of splicing graphs having alternative TTS events	691
Total number of splicing graphs having alternative initiation exon events	1,858
Total number of splicing graphs having alternative termination exon events	504
Total number of splicing graphs having alternative acceptor events	384
Total number of splicing graphs having alternative donor events	587
Total number of splicing graphs having cassette exon events	644
Total number of splicing graphs having intron retention events	700
Total number alternative TSS events	4,211
Total number alternative TTS events	1,511
Total number alternative initiation exon events	4,723
Total number alternative termination exon events	1,104
Total number alternative acceptor events	905
Total number alternative donor events	1,399
Total number alternative cassette exon events	1,228
Total number alternative intron retention events	983
